# A Shift in Conceptual Thinking of Panfacial Fracture Sequencing: The Major Fragment Theory

**DOI:** 10.3390/cmtr18010003

**Published:** 2025-01-03

**Authors:** Patrick Wong, Antonio Atte, David Powers, Paul Tiwana

**Affiliations:** 1 Division of Oral and Maxillofacial Surgery, Department of Surgery, UT Southwestern Medical Center, Dallas, TX 75390, USA; patrick.wong@utsouthwestern.edu; 2 Department of Oral & Maxillofacial Surgery, Health Sciences Center, The University of Oklahoma, Oklahoma City, OK 73104, USA; antonio-atte@ouhsc.edu; 3 Division of Plastic, Maxillofacial and Oral Surgery, Duke University Hospital, Durham, NC 27710, USA; david.powers@duke.edu

**Keywords:** trauma, maxillofacial trauma, panfacial fractures

## Abstract

Study Design: A literature review of relevant publications regarding panfacial fracture sequencing. Objective: To review the current landscape of sequencing of panfacial trauma and propose the utilization of the Major Fragment Theory when conventional sequencing techniques are inadequate. Methods: We conducted a review of existing literature on panfacial fracture management, focusing on sequencing techniques. Additionally, we analyzed unique fracture patterns to identify instances where conventional sequencing may be insufficient. Results: Existing literature emphasizes directional-based sequencing techniques for panfacial fracture reduction. However, unique fracture patterns often necessitate deviation from these sequences. The Major Fragment Theory suggests prioritizing the reduction of larger fragments over conventional sequencing, particularly when dealing with complex fractures. Conclusions: While directional-based sequencing techniques provide a valuable framework for panfacial fracture management and almost any approach can be utilized successfully, the Major Fragment Theory offers a complementary approach for cases where conventional sequencing falls short. Incorporating this theory into practice may enhance outcomes in the treatment of panfacial fractures.

## 1. Introduction

Though there is no universally accepted definition of panfacial trauma, in its simplest form, a panfacial fracture is defined as concomitant fractures in the upper, middle, and lower thirds of the face. These fractures are often secondary to high-speed blunt, penetrating, or avulsive mechanisms of injury, with motor vehicle collisions representing the majority [1,2,3]. Consequently, these patients often present with multisystem trauma, necessitating complex multidisciplinary care. Concomitant life-threatening injuries may affect surgical timing and further complicate the management of these facial injuries [4]. Panfacial fracture management aims to restore the patient’s face and function as close to their premorbid condition as possible and avoid any long-term sequelae caused by suboptimal correction.

Panfacial fractures pose a technical challenge to many facial surgeons due to the extensive nature of the injuries, significant comminution of the segments, and loss of anatomical references for reduction. Because of these challenging circumstances, the sequence of repair of the various fractures is critical to adequate management, and obtaining adequate imaging in the form of computed tomography (CT) with three-dimensional (3D) reconstruction is imperative. Several direction-based schemas for sequencing panfacial fractures have been described in the literature, specifically bottom-up versus top-down or outside-in versus inside-out. Here, the authors will discuss the critical points in repair sequencing and bring forth a theory regarding an intuitive but underemphasized factor in managing these fractures.

The authors posit that while in most cases these direction-based sequences are excellent frameworks for achieving ideal restoration of the face, in specific fracture patterns, one should opt to repair the fractures with the largest volumetric area and more obvious anatomical references for reduction even if it lies out of the order of these direction-based schemes. The authors refer to this idea as the Major Fragment Theory. This theory applies equally and effectively irrespective of which sequence the surgeon chooses for their particular approach to surgical treatment sequencing of these challenging fractures.

## 2. Methods

A PubMed search was performed with no date restrictions for literature on the management of panfacial fractures. The search terms used were “pan facial fractures”, “sequencing”, and various combinations of the terms. Articles not written in the English language were excluded.

## 3. Results

### 3.1. Anatomical Considerations

To effectively treat panfacial fractures, the surgeon must have an in-depth understanding of the anatomy of the facial buttresses, dental occlusion, and common fracture patterns.

**Figure 1:**
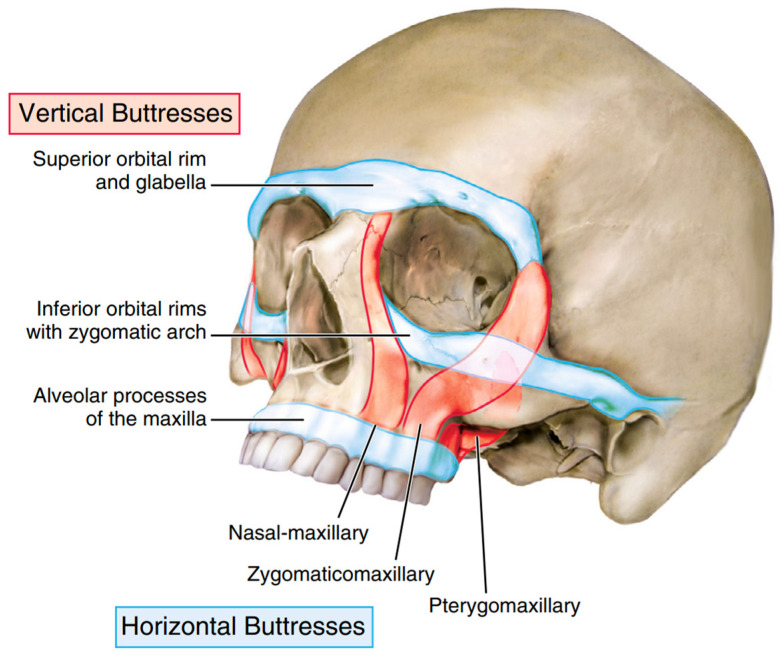
Buttresses of the Facial Skeleton (adapted from Atlas of Oral and Maxillofacial Surgery, Tiwana and Kademani).

Knowledge of the facial buttresses is paramount to optimal treatment of panfacial trauma. There are three paired vertical buttresses (nasomaxillary, zygomaticomaxillary, and pterygomaxillary) and three horizontal buttresses (the alveolar process of maxilla, inferior orbital rim/zygomatic arches, and superior orbital rim/glabella) (Figure 1). These buttresses are areas of thicker bone that transmit force and provide a framework for constructing facial function and form [5]. As such, they provide vital points of rigid fixation and anatomic reduction when treating these complex injuries. Adequate reduction of the buttresses lends to the return of premorbid facial form and stability ensuing repair [6]. Buttresses that have lost bony support may require immediate bone grafting.

Intimate knowledge of dental occlusion is also vital to restore both form and function to the dentate trauma patient. Restoration of adequate dental arch form is crucial to correcting lower facial width changes and facilitating the vertical positioning of the maxillomandibular complex. It is up to the clinician to assess the patient’s dentition to try and replicate their premorbid occlusion. Though this may prove relatively straightforward in a patient with a full complement of well-aligned teeth, significant difficulties may arise in patients with pre-existing malocclusions, partial dentition, or dentoalveolar and palatal fractures. These scenarios often require dental impressions and models to evaluate the dentition via hand articulation. This may subsequently require dental model segmentation and reduction, fabrication of splints, and strategic planning of dental extractions to achieve an optimal result. The advent of virtual surgical planning (VSP) has provided a digital means of achieving this.

### 3.2. Sequencing

#### 3.2.1. Initial Stabilization

Before the facial surgeon undertakes any surgical interventions, the patient is stabilized using Advanced Trauma Life Support protocols. For patients with compromised airways due to significant maxillofacial injury or decreased neurological status, a definitive airway should be established immediately. Severe injuries involving the brain, spine, thorax, and abdomen must be ruled out or adequately addressed before the patient undergoes any surgical correction of facial fractures [7]. Although ideally treated in an acute setting, the nature of these patients’ concomitant injuries often delay treatment by a few days or even sometimes weeks after their initial injury [4].

#### 3.2.2. Airway Management

Once the patient has been adequately resuscitated and deemed stable enough to undergo operative intervention for their facial injuries, a decision must be made on the appropriate airway management for the patient. A tracheostomy may be indicated if a prolonged course of mechanical ventilation is anticipated or the patient is expected to have multiple trips to the operating room. A tracheostomy allows for unimpeded access to all aspects of the face and does not interfere with maxillomandibular fixation (MMF) application.

Oral intubation is generally not feasible due to the necessity of placing the patient in MMF intraoperatively. However, in some instances, large edentulous gaps may allow for the tube to be placed around the dentition. Nasal intubation is also often not feasible in the context of panfacial fractures, as there is a possible risk of intracranial intubation if the patient has significant fractures of the skull base.

**Figure 2:**
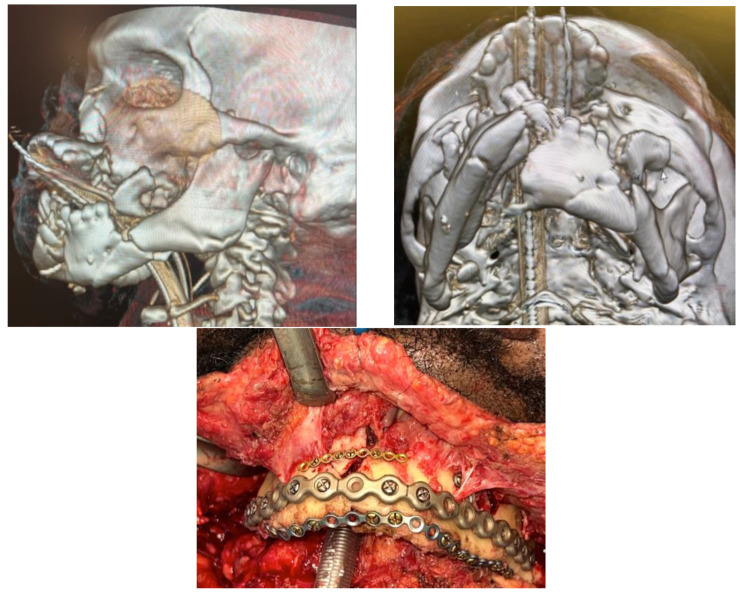
(**Upper Right** and **Left**): 3D reconstruction of a high-speed blunt injury with significant comminution of the maxillomandibular complex. (**Bottom**): Intraoperative photo of the mandibular fixation. Simplification plates can be used to piece together the comminuted pieces of the mandible. Submental intubation is a valuable form of airway management in patients not requiring tracheostomy.

A submental pull-through is a useful airway adjunct in cases where the patient will not require prolonged intubation postoperatively. It allows for the application of MMF without hindering access to most regions of the face save for extraoral approaches to the mandibular symphysis (Figure 2).

#### 3.2.3. Exposure of All Fractures

Once a decision has been made regarding the appropriate airway for the operation, the fractures to be treated via an open approach are exposed. Existing lacerations in advantageous locations can be utilized for simplified access.

A coronal incision is usually required to expose the frontal sinus, superior orbits, zygomatic arches, and central midface. This broad access is critical in cases where bone grafting of the upper midface may be required. Extraoral approaches to the mandible are generally preferred in cases of bilateral condylar fractures with a symphyseal component to visualize the lingual cortex of the mandible and prevent unwanted facial widening of the lower third. The nasomaxillary and zygomaticomaxillary buttresses and LeFort fractures can be accessed via a maxillary vestibular incision. Access to the orbital floors is usually a matter of the surgeon’s preference. The authors prefer a transconjunctival approach with a lateral canthotomy and inferior cantholysis. If needed, additional medial exposure may be achieved via a transcaruncular extension.

#### 3.2.4. Directional-Based Approaches to Sequencing

The literature is replete with information regarding directionally based approaches to sequencing, such as top-down, bottom-up, inside-out, or outside-in. The choice of which approach to use is generally up to the surgeon’s preference. However, most agree that no one approach is the ideal choice in every situation, and the surgeon must often tailor their sequence to the specific case in front of them.

##### Top-Down Approach

As Gruss and colleagues advocate, the top-down approach uses the orbital bar as the initial starting point for establishing the upper facial width [8]. This is based on the notion that the frontal-orbital bandeau is comprised of thick, dense bone that is not prone to gross comminution [9]. This leads to more significant fracture segments that are usually easier to reduce. Once the frontal sinus has been appropriately reduced, the anterior table of the frontal sinus and frontal-orbital bandeau can serve as a reliable starting point in all but the most severe panfacial fractures. The reduction continues to the zygomatic arches and zygomaticofrontal and zygomaticosphenoid junctions. The zygomas are then fixated at the zygomaticofrontal and zygomaticotemporal articulations, establishing both the facial width and midfacial projection. Next, the central midface and any naso-orbital-ethmoidal (NOE) fractures are addressed. It is typical in patients with panfacial fractures for significant facial flattening, loss of central projection, and comminution of the segments in this region. The vertical height of the face is then established by fixating the maxilla at the Lefort I level across the nasomaxillary and zygomaticomaxillary buttresses. The maxilla is then used as a reference to reduce and fixate the mandible. Alternatively, the mandible can be treated via closed reduction with MMF if the mandible has fractures involving the condylar processes that are not indicated for open reduction internal fixation (ORIF). The flexibility in treating the mandible closed is a proposed benefit of the top-down approach [8]. The top-down approach has been the historical choice for many surgeons, especially in cases of severe mandibular comminution or avulsion [10].

##### Bottom-Up

**Figure 3:**
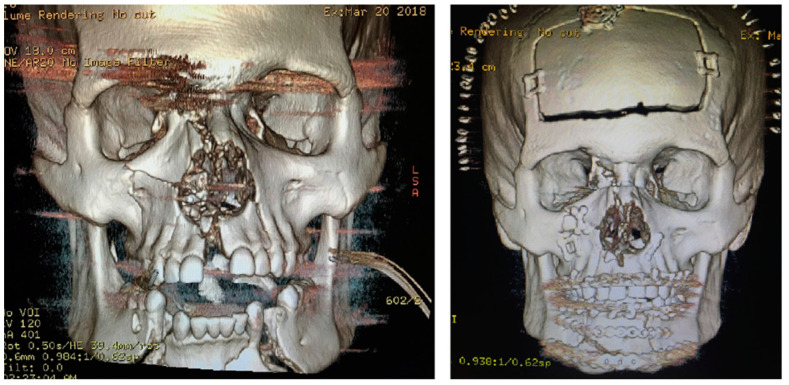
A case treated by the bottom-up, top-down, outside-in approach.

Advocated by Manson and colleagues, the bottom-up approach uses the mandible instead of the frontal-orbital bandeau as the foundation for the reduction sequence (Figure 3) [11]. This technique is often the standard choice of many Oral and Maxillofacial Surgeons [10]. This is likely due to the intimate familiarity of the specialty with the management of mandible fractures and the fact the mandible generally fractures into larger segments due to the quality of the bone stock, which allows for a more predictable establishment of a stable foundation for facial reconstruction. Any dentoalveolar fractures are reduced and fixated if necessary. The mandible is treated via open approaches to establish the lower face’s vertical height and an occlusal reference for the opposing maxilla. In the case of bilateral condylar fractures, ideally, both will be treated with open reduction internal fixation; however, in some cases, restoring a single condyle can be sufficient to regain the vertical height of the lower face.

**Figure 4:**
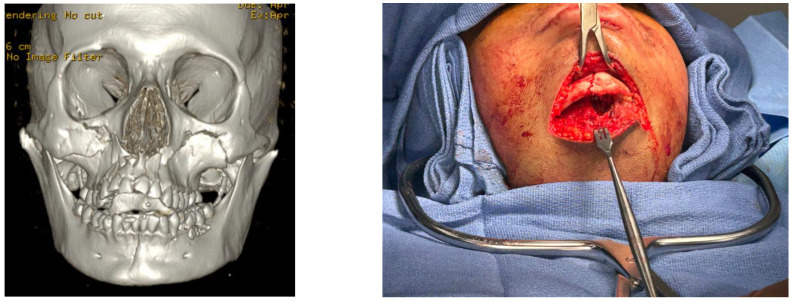
(**Left**): A patient with a LeFort I fracture and concomitant guardsman fracture with significant mandibular widening. Note that both condylar necks have been displaced laterally to the zygomatic arches. (**Right**): Acetabular clamp aiding in reducing the mandibular widening. Towel padding is used to prevent inadvertent facial injury.

In the case of significant segmentation of the maxilla secondary to dentoalveolar or palatal fractures, the mandible can be reduced anatomically, or a palatal splint fabricated using analog impressions or virtual surgical planning to recapture the transverse width of the dental arches. Often, the patient with a panfacial injury will have a symphyseal or parasymphyseal fracture with bilateral condylar fractures. This so-called “guardsman” fracture is prone to significant widening if splaying at the lingual cortex of the symphyseal fracture and the gonial width is not maintained. Transcervical access with a direct view of the lingual cortex during reduction and pressure at the gonial angles with either manual pressure or an acetabular clamp can help prevent this unwanted widening (Figure 4). If inadequately reduced, this can lead to a cascade effect, as the mandible is the foundation for the maxillary width in the bottom-up approach.

Once the mandible has been fixated, the resultant mandibular occlusion can be used as a reference for the maxilla, and the patient is placed into MMF. The bottom-up sequence described by Manson and Kelly as the “bottom-to-top-to-middle” approach then proceeds to the upper face and zygomas [12]. Correct placement of the zygoma position establishes the midfacial width and projection. With the zygomas positioned, the maxillomandibular complex can then be rotated superiorly to meet the skull base, and the zygomaticomaxillary buttresses can be used as a reference to position the complex, re-establishing the vertical height of the face. It should be noted that with the patient in the supine position, there may be a tendency for the entire maxillomandibular complex to rotate in a clockwise fashion. Therefore, care should be taken to seat the condyles properly and rotate the complex appropriately to restore the vertical and anteroposterior projection of the lower third of the face. Ideally, the reduction at the Lefort I level should be anatomic. However, some posit that minor discrepancies in alignment at the Lefort I level are of minimal aesthetic consequence provided the occlusion is satisfactory and the condyles are adequately seated [13]. This is similar to fixating the maxilla during a LeFort I osteotomy.

With the vertical height, width, and malar projection established, attention can be turned towards the central midface. The central midface often has the highest comminution in panfacial fractures. The highly comminuted segments of the central midface offer little reference for reduction and, for that reason, are addressed last. In many cases following reduction and fixation of the naso-orbito-ethmoidal (NOE) complex fractures, medial canthal tendon repositioning with transnasal wiring or cantilevered dorsal strut grafts are necessary to reproject the central midface.

##### Outside-In vs. Inside-Out

As mentioned earlier, the central midface is often the most comminuted region of the face in panfacial fractures. The thinner bones of this region, paired with the degree of comminution, make segment reduction more complicated than the more robust bony segments of the zygoma. For that reason, the authors employ an outside-in approach rather than an inside-out approach (Table 1).

#### 3.2.5. Major Fragment Theory

Much of the literature regarding the sequencing of panfacial fractures gives a preference for a specific directional-based approach to be used as a framework for sequencing these difficult injuries, with the caveat that the surgeon must tailor his or her approach to the pattern and severity of the fractures. Despite being a proponent of a bottom-up approach, Manson stated in 1999 that “any [order of treatment] is satisfactory if one understands the anatomy, goals, and procedures” [14]. This underscores the idea that, on occasion, the surgeon can violate the usual sequence of repair dictated by their directionally based frameworks with regard to sequencing. While this may be viewed by some in the context of the general surgical principle of “known to unknown” in the performance of operations, which has been taught as a tenet of treatment in all surgical disciplines and is not specific to craniomaxillofacial surgery nor has it been applied to complex surgical sequencing for facial fracture management. The Major Fragment Theory attempts to further elucidate this general principle as it specifically relates to panfacial fracture management, which has traditionally followed a set pattern of steps and has not been formally explored in the literature previously. Freedom from the rigidity of a tightly sequenced plan, regardless of which one is selected, can allow for the efficient treatment of fractures and further optimize patient outcomes.

The Major Fragment Theory is simply described as being more beneficial in reducing and fixating large fracture segments with minimal comminution and clinically evident landmarks earlier in surgery, regardless of where that segment lies in the usual directionally based sequencing framework. These larger segments with obvious reductions are the “known” territory that should be established before addressing areas with higher degrees of comminution or avulsion, which constitute the “unknown” territory.

Panfacial fractures are often compared to a jigsaw puzzle with several pieces in the literature [15]. When solving a puzzle, it is generally easier to establish the pieces on the borders and then methodically work towards the center. This concept is analogous to the directionally based approaches of sequencing. However, apparent patterns or pictures printed on the puzzle pieces can be used as easy landmarks for assembly even if those pieces lie outside the periphery or are “out of order.” This provides a succinct illustration of the concept of the major fragment theory. Facial trauma surgeons should have both concepts in mind and not adhere rigidly to a directionally based approach when sequencing panfacial fractures.

### 3.3. Case Example

**Figure 5:**
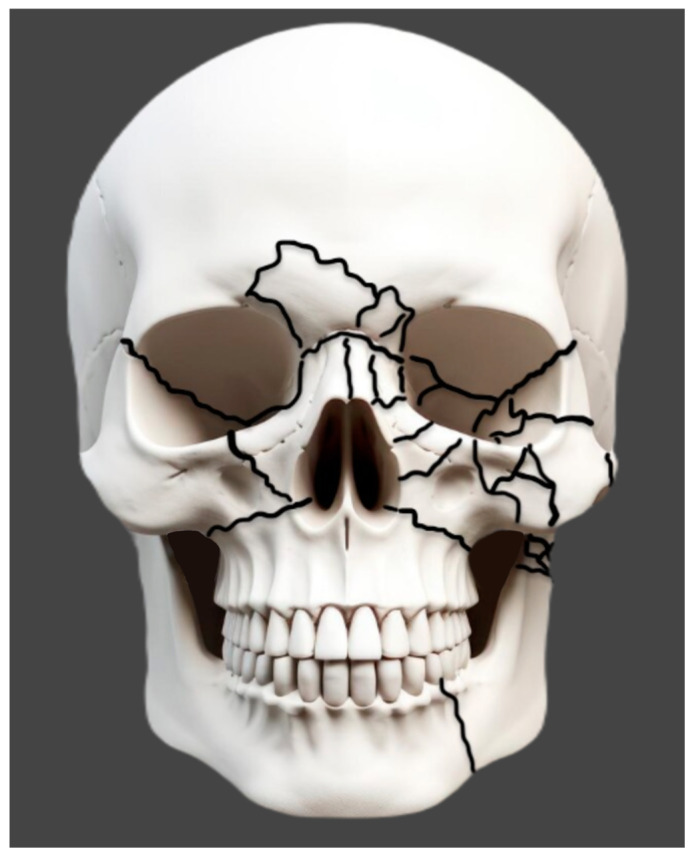
A hypothetical panfacial Fracture to illustrate the implementation of the Major Fragment Theory.

An illustration of a hypothetical case where utilization of the Major Fragment Theory would be beneficial can be seen in Figure 5. In this case, there is significant comminution of the left mandibular condyle for which ORIF may not be feasible. Furthermore, the left midface is significantly comminuted with minimal anatomic structural references. In contrast, the right side has a simple zygomaticomaxillary complex (ZMC) with linear fractures and a Markowitz Type I NOE fracture. Following ORIF of the left body fracture, it may be beneficial to complete ORIF of the Right ZMC and NOE fractures to use as a reference to position the maxillomandibular complex once the patient has been placed into MMF before treating the left midface. This would allow for correcting the anterior-posterior and vertical positions of the midface and mandible in a more predictable sequence to reduce the fractures. By sequencing in this manner, the surgeon has violated the classical outside-in sequence but significantly simplified their surgical approach.

## Figures and Tables

**Table 1:** Surgical Sequencing of Panfacial Fractures with the Bottom-Up, Top-Down, Outside-In Approach.

Surgical Sequence for a Bottom-Up, Top-Down, Outside-In Approach
Establishment of continuous dental arches via reduction of dentoalveolar fractures, palatal splinting if necessary
Open Reduction Internal Fixation of Mandible fractures
Placement of the patient into Maxillomandibular Fixation
Management of frontal sinus and orbital roof fractures with appropriate cranialization, obliteration, ORIF, etc.
Open Reduction Internal Fixation of Zygoma Fractures
Rotation of the maxillomandibular complex and fixation at the Lefort I level
Repair of any indicated orbital floor or medial wall fractures
Addressing fractures of the central midface and NOE complex with necessary medial canthal tendon management and dorsal strut bone grafting

## Data Availability

No new data were created or analyzed in this study. Data sharing is not applicable to this article.
